# Usefulness of Cryoprobe in Office Hysteroscopy for Removal of Polyps and Myomas

**DOI:** 10.1155/2018/7104892

**Published:** 2018-08-26

**Authors:** Kamil Sobociński, Jacek Doniec, Magdalena Biela, Monika Szafarowska, Krzysztof Paśnik, Paweł Kamiński

**Affiliations:** ^1^Gynaecology and Oncological Gynaecology Department, Military Medical Institute, Szaserów 128, Warsaw 04-141, Poland; ^2^Department of General, Oncological, Metabolic and Thoracic Surgery, Military Medical Institute, Szaserów 128, Warsaw 04-141, Poland

## Abstract

Hysteroscopy is a gold standard in 21^st^-century gynaecology for both diagnosis and treatment procedures of intrauterine pathologies. Miniaturisation of the equipment and better techniques of performing this procedure allowed it to become the gold standard. Nevertheless, hysteroscopy has its limitations, which is the size of the endometrial polyps or submucous myomas. We have invented a new device for the 5Fr working channel hysteroscopes for possessing and resecting intrauterine structures: the cryoprobe. The retrospective cryobiopsy study presented here was conducted at the Department of Gynaecology and Oncological Gynaecology, Military Institute of Medicine in Warsaw, Poland, from October 2017 to January 2018. Its purpose was to assess the usefulness of the new device in office hysteroscopy for the removal of polyps and myomas with a diameter over 10 mm. Thirteen patients with an initial diagnosis of an endometrial polyp or submucous myoma were enrolled in the trial. All procedures took place in day-surgery settings, with a total resection of the pathological intrauterine structure, without complications. The application of the cryoprobe may enhance the usefulness of office hysteroscopy, without extending the procedure. The cryoprobe efficiency is still under research in a bigger group.

## 1. Introduction

Hysteroscopy, thanks to which in the 19th century it was first possible to see the inside of the uterus, is considered a milestone in the development of modern gynaecology. During the last 40 years, the dynamic development of endoscopic technologies has revolutionised the diagnostics of uterine cavity pathologies, thus enabling the performance of very precise, sight-controlled procedures, according to the principle “see and treat” [1]. Thanks to this, hysteroscopy has gained the status of a gold standard in the diagnostics and treatment of uterine cavity pathologies. Introducing “ambulatory hysteroscopy” into ambulatory practice, using hysteroscopes ≤5mm in diameter, of a continuous flow system, and designing hysteroscopic tools of a miniature size (scissors, graspers, vulsella, and forceps), allowed the performance of such diagnostic and surgical procedures as resecting polyps and myomas without the necessity of the patient's anaesthesia. The greatest limitation of these minimally invasive procedures is the size of the resected structure and the resulting difficulty in removing it from the uterine cavity. The method of fragmenting the pathological structure is extremely time-consuming, while using a hysteroscope of a greater diameter results in a greater invasiveness of the procedure and often requires anaesthesia. In many centres studies are being conducted in the search of new tools that would broaden the scope of possibilities of ambulatory hysteroscopy. At our clinic we are developing and testing a new tool that allows the resection of bigger endometrial polyps and submucous myomas that uses low temperature. The cryoprobe designed by our team is appropriate to be used with a hysteroscope of a diameter of 4-5mm with a working channel of 5Fr, and combines the functions of grasping and resecting thanks to the use of low temperatures at the tip of the tool. This invention broadens the scope of minimally invasive treatment of bigger polyps and myomas, without a dilatation of the cervical canal for the classic 9mm resectoscope or anaesthesia. The aim of the present paper is to present this new tool—the cryoprobe—and provide a preliminary assessment of its usefulness for resecting and removing polyps and myomas over 10mm in diameter from the uterine cavity.

## 2. Materials and Methods

**Figure 1:**
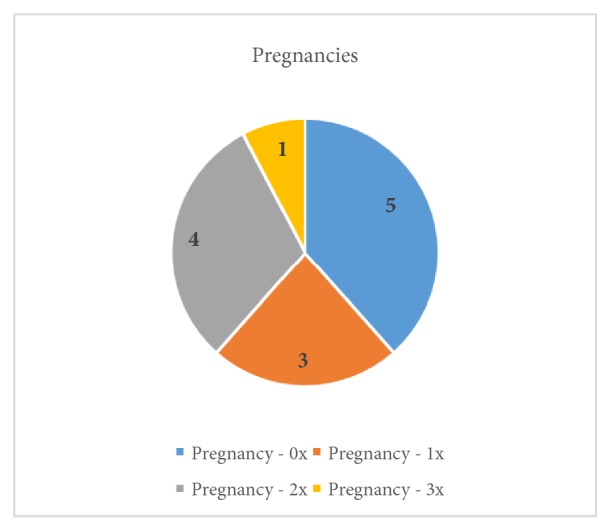
The patients' pregnancies.

**Figure 2:**
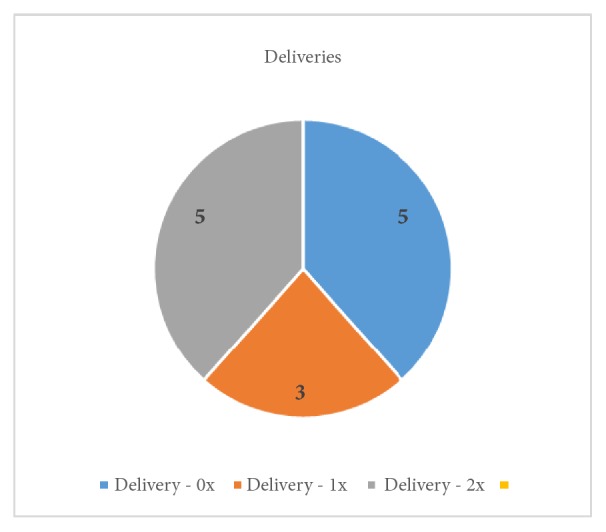
The patients' deliveries.

This retrospective cryobiopsy trial study was conducted at the Department of Gynaecology and Oncological Gynaecology, Military Institute of Medicine in Warsaw, Poland, in the period from October 2017 to January 2018, and was approved by the ethics committee of the hospital. At the department, 1200 hysteroscopies are performed a year. We enrolled patients aged 28 to 69 (mean age 44; SD: 11,45). The initial diagnosis of a uterine pathology was based on a transvaginal ultrasonography and clinical signs. In all patients, the exclusion criteria were pregnancy, cervix neoplasia, active PID, and active severe menstrual bleeding. The pregnancies and deliveries are shown in Figures 1 and 2. Two patients were after menopause, and for one of them the main indication for the procedure was abnormal uterine bleeding. Only the youngest (aged 28) had hormonal treatment (oral contraceptive pill). 9 out of 11 patients in the reproductive age suffered from excessive menstrual bleeding.

**Figure 3:**
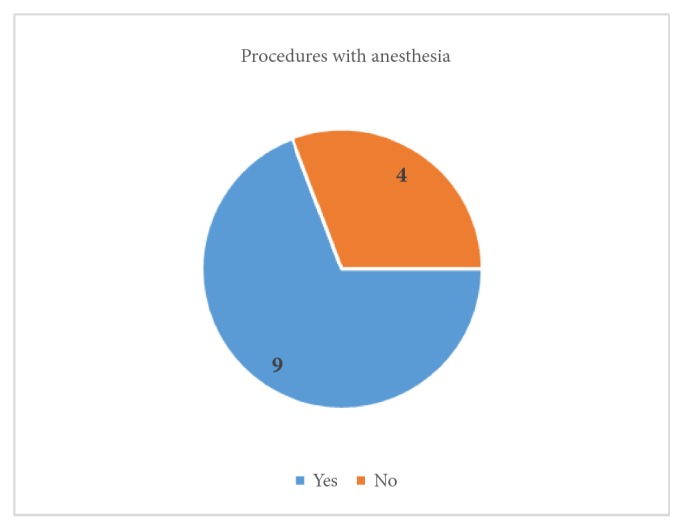
Procedures with anaesthesia.

In each patient, operative hysteroscopy was performed. The hysteroscopy was performed in the proliferative phase of the menstrual cycle; however, for patients after menopause the day was irrelevant. Depending on each patient's gynaecological conditions and preferences, the procedures were performed with or without anaesthesia; the proportions are presented in Figure 3.

**Figure 4:**
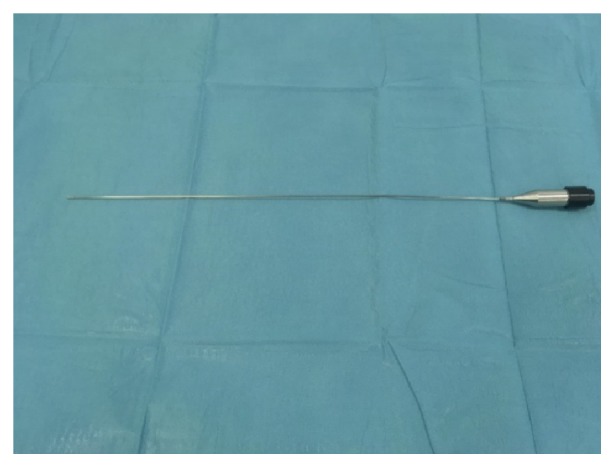
The cryoprobe.

The rigid hysteroscope, Karl Storz Endoscope, Germany, with an oval profile and a width of 4mm or 5mm, was used for all patients. The procedure was performed following aseptic rules, without a disinfection of the vagina nor a sterile cover, without using either a speculum or tenaculum forceps—according to prof. S. Bettocchi's method [2]. After a visual inspection, the pathological structure was resected with hysteroscopic tools—scissors, a grasper, or a bipolar electrode (Twizzle type, Gynecare). Conventionally, the resected structures were removed from the uterine cavity with a grasper, preserved in 4% formaldehyde and transferred to the pathology department for microscopic assessment. Usually, when the resected structure is above 10mm in diameter in the narrowest dimension, and a problem with removing it from the uterine cavity occurs, we use the cryoprobe. The cryoprobe (Figure 4) was designed based on an idea of Jacek Doniec, our gynaecologist. It is manufactured by a company with 25 years of experience in constructing and producing cryogenic devices used in gynaecology, dermatology, ophthalmology, oncology, pain management, and surgery. It has a 5Fr diameter and semiflexible straight probe and is 40cm long and compatible with the Carl Storz Hysteroscope; and it is very easy to use.

**Figure 5:**
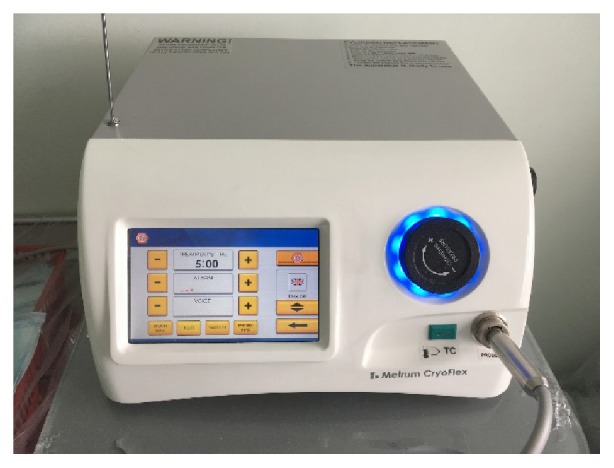
Metrum Cryoflex generator.

The cryoprobe has a CE mark in accordance with the ISO13485 norm. It works with every type of universal generator for cryotherapy manufactured by Metrum Cryflex (Figure 5). The probe can be steam sterilized (121°C).

The mechanism that enables the operation of the cryoprobe is that an adhesive force is created on the tip of the cryoprobe, according to the Joule-Thompson principle. Such cryobiopsy allows removing larger fragments of tissue from the uterine cavity, both hard and soft. A lower risk of bleeding due to the haemostatic features of cryotechnology is another asset here [3]. During the procedure, the pathological structure is attached to the pointy end of the electrode—either after the structure has been resected or without a resection. The adhesive force is managed by reducing the temperature to -70°C, only at the tip of the electrode. This adherence is created within a few seconds and lasts only during the freezing stage. After connecting the tissue to the probe, the hysteroscope is then removed with the probe and the attached sample from the uterine cavity. Polyps, due to their flexibility, usually squeeze easily through the cervical canal. However, when myomas are resected, the surgeon needs to use more force to remove the tissue from the uterine cavity. After a few seconds of the defrosting stage, the tissue is easily separated from the cryoprobe. If the removed structure is incomplete, the hysteroscope is put back into the uterine cavity and the procedure is repeated until the desired effect is achieved. In the present study, all procedures where the cryoprobe was used were performed by one endoscopist in the office setting during a day-surgery.

## 3. Results

Tables 1 and 2 shows the study results divided into two groups, according to the final diagnosis after the performed procedures.

In total, 13 patients were included in the trial. In 3 patients, the following submucosal myomas were resected with the cryoprobe during the operative hysteroscopy procedure:

- 1 submucosal myoma, 10mm in diameter (ultrasound and hysteroscopic assessment);
- 2 submucosal myomas, 5mm in diameter each, and an endometrial polyp;
- 1 submucosal myoma, 20mm in diameter in hysteroscopic assessment (16mm in ultrasound examination), primarily classified as an endometrial polyp.

**Figure 6:**
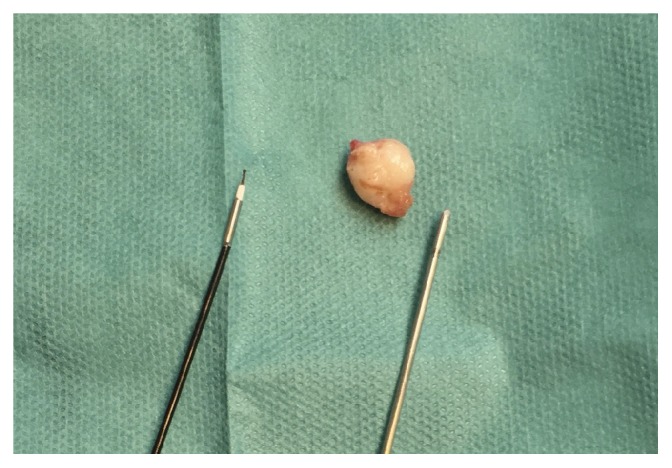
From the left: bipolar electrode, submucosal myoma, and cryoprobe.

Figure 6 shows a submucosal myoma, 10mm in diameter, with the cryoprobe on the right and the Twizzle bipolar electrode on the left, to compare. The myoma was enucleated and extracted from the uterine cavity in one piece, at a single attempt, only with the use of the cryoprobe.

The cryobiopsy method was also applied in 10 patients with an ultrasound finding that fulfilled the endometrial polypus criteria. The polyps were assessed as above: 10mm in diameter both in a transvaginal ultrasound and a hysteroscopic assessment. The widest polyp was 26×8×16mm in the ultrasound, and almost the same size in the endoscopic view. First, the polyps were resected with the Versapoint electrode. Next, due to the impossibility of evacuating the polyps using standard endoscopic equipment, the cryoprobe was used. In 9 out of 10 procedures, it was enough to perform a single attempt. Only in one patient the polyp was removed in fragments. In one case the structure diagnosed as a polyp during hysteroscopy was evaluated as a myoma in the pathological examination. All patients with an initial diagnosis of a myoma had the procedure performed in general anaesthesia. In four patients with a diagnosis of an endometrial polyp the procedure was carried out without anaesthesia; the discomfort was tolerated by the patient, and the pathological tissues were removed completely. All of the hysteroscopies took place as a one-day procedure and caused no complications, and the patients left the hospital on the same day as the procedure.

## 4. Discussion

Cryoenergy has been used in medicine for a long time. The standard application of it is to destroy pathological tissue, for example, in uterine cervix cryotherapy [4, 5] or in endoscopic spray cryotherapy for Barrett's oesophagus with dysplasia [6]. In 2009, the first bronchoscopic cryobiopsies were described as an alternative to the classical bronchoscopic biopsy with the use of forceps. Since then, several studies have been conducted, mostly with similar promising results in the diagnostic yield, complication rate, and safety. The basic principle in this method is to create a low temperature (around -70°C) at the end of the probe. During the freezing, a strong adhesive force is produced. Thus, tissue firmly adheres to the probe and is easy to collect. After a few seconds the probe defrosts, the connection disappears, and the sample is then separated from the end of the probe. Moreover, removing the tissue fragments using the cryoprobe provides a full hemostasis at the site where the tissue has been removed [7]. The safety of the cryoprobe has also been confirmed in the treatment of varicose veins in the shank—the cryostripping technique [8]. Using low temperature at cryostripping of varicose veins generates a force that is sufficient to remove a vein vessel; moreover, it also provides a hemostasis of the collateral vessels. Combining and adapting these benefits, we have created the first tool to be used in minihysteroscopy that allows pathological structures of a diameter over 10mm to be resected in ambulatory conditions.

Minihysteroscopy as a method that is a gold standard in diagnostics and treatment of uterine cavity pathologies unfortunately also has its limitations [9]. The available tools that are compatible with the hysteroscope with a working channel of 5Fr often make the removal of bigger pathologies from the uterine cavity impossible. For medium and large endometrial polyps and small submucous myomas (below 20mm), the “slicing technique” allows a resection of the pathology; however, it is very time-consuming and requires experience. In such cases, according to the recommendations, larger pathological structures should be removed using a resectoscope [9]. In order to broaden the scope of possible procedures in ambulatory minihysteroscopy, we proposed a new tool using cryoenergy, described in the present paper.

As mentioned above, the use of a cryoprobe in hysteroscopy is a solution that enables a resection of larger pathological structures, yet maintaining the minimal invasiveness of hysteroscopy. The adhesive force created by the cryoprobe is sufficient to overcome the resistance of the cervix and remove the resected tissue from the uterine cavity. Moreover, with small submucous myomas, the cryoprobe allows them to be totally enucleated from the uterus wall. It is a less invasive alternative to the classical cervix dilatation and using the 9mm resectoscope for small myomas; it is also easier and faster than enucleating them with the standard bipolar Twizzle tip electrode. The minimal invasiveness of the procedure is especially crucial for patients at a reproductive age, especially those who are treated due to infertility.

In the case of performing the procedure without a general anaesthesia, using the cryoprobe allows the procedure to last significantly shorter. What follows, the patient's discomfort related to the procedure itself is thus reduced, and the risk of complications is lower. None of the patients who had the procedure carried out without a general anaesthesia reported strong pain discomfort, weakness, or dizziness after the procedure. The discomfort reported by the patients was typical for the procedure and was fully accepted by them. Thus, in an attempt to minimise the invasiveness of the procedures, the cryoprobe may prove to be useful in office hysteroscopy [10].

The histopathological assessment of the material collected using the cryoprobe took place according to the standard histopathological schemas. During the analysis, no faults in the quality of the samples provided were reported or damage of the tissue other than with standard hysteroscopy. The material collected using the cryoprobe was frozen only for about a dozen seconds, and then it was preserved in a formalin solution and transferred for routine histopathological assessment. The doctors examining the samples were not informed about the use of the cryoprobe. Intraoperative examinations are commonly carried out using the method of freezing the samples, with a proven high sensitivity and specificity [11]. However, the possibility and the quality of a histopathological examination of samples collected using a cryoprobe require further studies.

The progress in minimally invasive surgery using modern technologies allows a treatment of patients that is almost nontraumatic, in ambulatory conditions. In gynaecology, especially in reproductive medicine, minihysteroscopy plays a crucial role. Many studies have shown that the presence of endometrial polyps or submucous myomas in the uterine cavity is a cause of pathological uterine bleeding, infertility, and obstetrical complications. [12]. Therefore, resecting these pathological structures, regardless of their size, in a way that is possibly least invasive, not traumatic for the cervix, and above all, safe, is especially crucial. The cryoprobe created by our team fills this technological gap, thus enabling a resection of large pathological structures from the uterine cavity.

## 5. Conclusions

- The cryoprobe used during ambulatory hysteroscopy may broaden the scope of possibilities of this technique by facilitating the resection of submucous myomas and endometrial polyps of a diameter above 10mm from the uterine cavity and may shorten the time of the procedure.
- Further studies in a bigger group are required on the safety and effectiveness of using the cryoprobe for hysteroscopic procedures.

## Figures and Tables

**Table 1:** Group of patients with endometrial polyps.

USG assessment	26×8×16 mm	10×7 mm	20×8 mm	12×8 mm	18 mm diameter	20 mm diameter	25×10 mm	15×9×20 mm	18×14 mm

Hysteroscopic assessment	25×15 mm	20 mm diameter	20×10 mm	15mm, 5mm diameter each	20 mm diameter	25 mm diameter	15mm, 10mm diameter	15×10 mm	15 mm diameter

Final diagnosis	Endometrial polyp	Endometrial polyp	Multiple endometrial polyps	Multiple endometrial polyps	Endometrial polyp	Endometrial polyp	Multiple endometrial polyps	Endometrial polyp	Endometrial polyp & cervical polyp

**Table 2:** Group of patients with submucous myomas.

USG assessment	10×8×9 mm	16 mm diameter	14×8 mm	15×16 mm

Hysteroscopic assessment	10 mm diameter	20 mm diameter	10 mm diameter	10mm, 5mm, 4mm diameter each

Final diagnosis	Submucous myoma	Submucous myoma	Submucous myoma	Submucous myoma, endometrial polyp

## Data Availability

The data used to support the findings of this study are available from the corresponding author upon request.
